# Milk profiles of selenoamino acids, selenoenzymes, and selenoproteins of peripartal dairy cows fed with different selenium sources

**DOI:** 10.5713/ab.24.0571

**Published:** 2025-01-24

**Authors:** Xiaoyu Ji, Xuejuan Deng, Ning Liu, Jianping Wang

**Affiliations:** 1 Department of Animal Science, Henan University of Science and Technology, Luoyang, China; 2 Department of Research and Development, National Engineering Research Center of Biological Feed, Beijing, China

**Keywords:** Dairy Cows, Nanoselenium, Selenoamino Acids, Selenoenzymes, Selenoproteins

## Abstract

**Objective:**

This study aimed to investigate the effect of dietary nanoselenium (nanoSe) and sodium selenite (SS) on the concentrations of selenoamino acids, the activities of selenoenzymes, and the mRNA expressions of selenoproteins in the milk of peripartal dairy cows.

**Methods:**

Three diets included a control (basal diet) with background selenium at 0.06 mg/kg and treatments with either SS or nanoSe added at the same selenium concentration of 1.00 mg/kg of diet. A total of 45 dairy cows were randomly allocated to three groups. The feeding trial lasted for 42 days from prenatal 21 days to postnatal 21 days.

**Results:**

NanoSe increased (p<0.05) milk yield compared to the control and SS. In milk, both SS and nanoSe increased (p<0.05) the concentrations of selenium, selenocysteine, and selenomethionine, and the activities of glutathione peroxidase 4, glutathione reductase, thioredoxin reductase, glutathione S-transferase, and iodothyronine deiodinases (type 2 and 3). The nanoSe showed higher (p<0.05) effects on these parameters than SS. Also, dietary nanoSe upregulated (p<0.05) the mRNA expressions of selenoproteins P, W, S, F, M, N, K, O, H, and I in milk compared to the control. For most selenoproteins, there was no difference between SS and nanoSe, only selenoprotein K was higher (p<0.05) in nanoSe than in SS.

**Conclusion:**

In conclusion, dietary nanoSe increased milk yield, milk selenoamino acids and selenoproteins in peripartal dairy cows.

## INTRODUCTION

Selenium is an essential trace element that plays a crucial role in various biological processes, including antioxidant defense and thyroid hormone metabolism [1]. Selenium acts mainly in the form of selenoamino acids, which are a unique group of amino acids that contain the element selenium in place of sulfur [2]. Selenoamino acids mainly include selenocysteine (Sec) and selenomethionine (SeMet), and the former is of particular importance for the synthesis of selenoproteins including selenoenzymes. Selenoamino acids can be naturally found in foods such as Brazil nuts, seafood, and organ meat; and in most cases, they are at a background content in the diet can act as a nutritious boost to support overall health and well-being [1]. However, the deficiency of dietary selenium is lethal under the various stresses from physiology or the environment, especially in farm animals with high productivity [3,4].

Selenoenzymes are one of the most important parts of selenoproteins and play crucial roles in various biological processes, including antioxidant defense, thyroid hormone metabolism, and immune function [5]. One of the most well-known selenoenzymes is glutathione peroxidase (GPx), which helps protect cells from damage caused by reactive oxygen species. Another important selenoenzyme is iodothyronine deiodinase (DIO), which is involved in the activation and deactivation of thyroid hormones [6]. Besides, other identified selenoenzymes, such as thioredoxin reductase (TrxR), glutathione reductase (GR), and glutathione S-transferase (GST), perform similar functions to GPx in maintaining proper physiological functions [5,7].

The selenization in the body is largely dependent on the sources and concentration of dietary selenium and the species and physiological status of farm animals [8]. Nano selenium (nanoSe) refers to the chemical, physical or biological method of reducing selenium ions to elemental selenium that group together on a nanoscale [8–10]. Recent studies showed that nanoSe is more bioavailable and safer than other forms [9,10]. Han et al [11] reported that dietary supplementation with nanoSe at 0.3 mg/kg had no effects on dry matter intake (DMI), milk yield and composition, but increased milk selenium and GPx activity, and upregulated the mRNA expression levels of GPx (1, 2 and 4), TrxR (2 and 3), and selenoproteins (W, T, K and F) in the mammary gland of mid-lactation dairy cows. The findings in the literature indicated that dietary nanoSe stimulated the antioxidant status and selenoprotein gene expressions in the mammary glands of dairy cows.

The peripartal period is a key stage in the lactation cycle of dairy cows. Calving and lactation pressure may cause some physiological and metabolic dysfunction, threatening health and efficient production of dairy cows. Given the limited information on the effects of dietary nanoSe on selenoamino acids, selenoenzymes, and selenoproteins in dairy cows, this study aimed to investigate the effect of nanoSe on the milk production, selenoamino acids, selenoenzymes, and selenoproteins in dairy cows during the peripartal period.

## MATERIALS AND METHODS

### Animal ethics

The feeding trial and sample collection in this study were approved by the Animal Care Community of Henan University of Science and Technology (#2023065, Luoyang, Henan, China).

### NanoSelenium and diets

The preparation and characterization of nanoSe were referred to the method by Al-Quwaie [12] with minor modifications. Briefly, *Bacillus Subtilis* (No. CGMCC 13131) from China General Microbiological Culture Collection Center (Beijing, China) was inoculated into Luria-Bertani agar medium, and single colonies were picked and cultured in a bacterial culture flask filled with Luria-Bertani liquid medium (100 μL) at 37°C and 180 rpm for 24 h. The mixture was centrifuged for 20 min at 4,500 rpm. The supernatant was obtained, then mixed with 100 mL of Luria-Bertani broth supplemented with 4.85 mmol/L sodium selenite (SS), and cultured at 37°C and 180 rpm for 24 h. With the incubation, the color change from yellow to red demonstrated the transformation of SS to nanoSe. The culture liquid was centrifuged at 4,500 r/min for 3 min to obtain the precipitate with nanoSe. The precipitate was rinsed with distilled water for 3 times to discard SS. At last, the precipitate was dried at 65°C and nanoSe powder was obtained. The concentration of nanoSe in the final product powder was 79 mg/g. The bacterial reduced nanoSe possessed a size of 15 nm determined by Zetasizer (Nano Z; Malvern Panalytical, Malvern, UK).

There were three diets including a control (basal diet, total mixed ration [TMR]), background selenium 0.06 mg/kg and diets with either supplemental SS (containing selenium at 1.0 mg/kg of diet) or nanoSe (1.0 mg/kg of diet). The determined levels of selenium in the diets of SS and nanoSe were 1.04 and 1.02 mg/kg, respectively. All dietary determination were carried out at dry matter (DM) basis. The nutritive requirements of the basal diet were referred to the NRC and with minor modification according to the practice of the dairy farm. The diet ingredient composition and nutrient levels are listed in Table 1.

### Animals and samples

A total of 45 Holstein dairy cows without statistical differences (mean±SD) in parity (2.3±0.9), milk yield in the last lactation (28.5±1.4 kg/d), body weight (651±47 kg), and differences in expected delivery date (±3.5 d) were randomly allocated to three groups of 15 replicates each according to a complete randomized design. The cows were housed in the tie stalls at the Shengsheng Dairy Cattle Co. (Luoyang, China) with *ad libitum* access to water and TMR. Cows were fed at 08:00, 15:00, and 22:00 h, and milked after delivery at 07:00, 14:00, and 21:00 h. The feeding trial after a week adaptation lasted for 6 weeks covering both prenatal 3 weeks and postnatal 3 weeks.

In the morning, noon, and evening on day 14 and 7 before delivery, and day 7, 14 and 21 after delivery, feed consumption and leftovers were recorded. Meanwhile, feed samples were collected using a four-point method for determining DM content and calculating DMI. Milk production of cows was recorded four times every 7 days postpartum with a one-day interval. On day 7, 14, and 21 postpartum, 50 mL of milk samples were collected in the morning, noon and evening, and mixed by the day in the percentages of 40%, 30%, 30%, respectively, and stored at −80°C. Appropriate 100 mL of milk samples were used to determine milk composition with a fully automated milk composition analyzer (MilkoScan-FT120; FOSS, Hillerød, Denmark) and 4% fat-corrected milk (FCM) production was calculated; others were prepared for the quantification of milk selenium, selenoamino acids, selenoenzymes, and selenoproteins.

### Chemical and biochemical analysis

Selenium in samples was quantified using a Target Selenium Kit (#abx298910; Abbexa LTD., Cambridge, UK) by colorimetric method. The contents of Sec and SeMet in the milk were analyzed using an HPLC system (Waters 2489; Waters, Milford, MA, USA) according to the method by Bierla et al [13] with modifications by Sun et al [14]. The activities of selenoenzymes were analyzed using commercial kits from Nanjing Jiancheng Biological Institute (Nanjing, China) for GPx4 (H545-1-1), TrxR (A119-1-1), GR (A062-1-1), and GST (A004-1-1). Assay kits of ELISA from BioVenic Co. (Hauppauge, NY, USA) were used for the determination of bovine DIO2 (IVEK1311) and bovine DIO3 (IVEK2335). One sample was detected three times.

Total RNA was isolated from milk samples, and was used to synthesize cDNA with random hexamers and RNase inhibitors in the reaction. A control without reverse transcriptase was used to check the genomic DNA contamination. The mRNA level was expressed as 2^−ΔΔCt^ relative to a house-keeping gene [15]. Primer information is listed in Table 2. SYBR Green Master Mix was used for quantitative polymerase chain reaction (qPCR) reactions by ABI PRISM®7900H of Applied Biosystems (Foster City, CA, USA). The qPCR was set at 10 μL with SYBR (5 μL), primer (1 μL), and 10×diluted cDNA (4 μL) under conditions of 40 cycles: denaturation (3 min, 95°C), annealing (15 s, 95°C), and extension (1 min, 60°C). Kits for real-time-qPCR analysis and primer synthesis were provided by TaKaRa Co. (Dalian, China).

### Statistics

Data are represented as covariate-adjusted least square means and standard error of the mean by the Proc Mixed procedures of SAS (version 9.4; SAS Institute Inc., Cary, NC, USA) with an autoregressive covariance structure. The model analysis includes fixed effects (treatment and week), interaction (treatment×week), and random effect (cow). Differences between the mean values were assessed by Tukey’b-test at a significance level of p<0.05.

## RESULTS

### Milk production

NanoSe increased (p*≤*0.027) milk yield, milk protein, milk yield/DMI, and FCM compared to control (Table 3). Also, nanoSe increased (p<0.05) milk yield and FCM compared to SS. There were interactions (p*≤*0.041) between treatments and weeks on milk yield, milk yield/DMI, milk fat, milk total solids, and FCM.

### Milk selenium, selenoamino acids, and selenoenzymes

Both nanoSe and SS increased (p<0.001) the contents of selenium, Sec, and SeMet, and the activities of GPx4, GR, TrxR, GST, DIO2, and DIO3 in milk compared to control (Table 4). nanoSe showed greater effects (p<0.05) on these parameters than SS.

### The mRNA expression of selenoproteins

NanoSe and SS upregulated (p<0.001) the mRNA levels of selenoP, selenoW, selenoS, selenoM, selenoN, selenoK, selenoO, selenoH, and selenoI compared to control (Table 5). NanoSe upregulated (p<0.05) selenoT expression more than control, and selenoK more than SS.

## DISCUSSION

In this study, dairy cows supplemented with nanoSe during the peripartal period showed a significant increase in milk yield, indicating the effect of nanoSe on the performance of dairy cows. Liu et al [16] reported that nanoSe supplemented at 0.1, 0.2, and 0.3 mg/kg increased milk production by promoting rumen fermentation, nutrient digestion, and mammary gland development. The antioxidation effect of dietary selenium is well known [8,9], so this study avoided repeating, and the antioxidation of dietary nanoSe and its cascades on selenoamino acids, selenoenzymes, and selenoproteins may be the reason for increased milk production. In cattle, it is unclear how dietary selenium supplementation affects fetal development. In other animals, adding dietary nanoSe (0.5 mg/kg) to goat gestation diets promoted fetal growth [17] and in sows dietary selenium supplementation increased litter weight gain and selenium profiles [18,19]. In this study, the effect of nanoSe on fetuses and calves was not determined, which deserves further study. Additionally, most milk components changed with the progress of lactation time, which caused interactions between dietary treatments and lactation weeks.

Both SS and nanoSe supplementation significantly increased the content of selenium and selenoamino acids in milk. However, the effect of nanoSe was more pronounced, indicating its higher bioavailability and deposition in milk. This is consistent with previous reports that nanoSe was more readily absorbed and utilized by animals compared to SS [11,20]. The increased contents of selenoamino acids including SeMet and Sec in milk are particularly important as they help improve dairy products’ overall nutritional value by providing consumers with selenium [5,9]. Moreover, the Sec is the crucial component of selenoproteins including selenoenzymes which play a pivotal role in antioxidant defense, thyroid hormone metabolism, and immune function [21]. The increased concentration of these selenoamino acids in milk suggests that nanoSe supplementation has a positive effect on the synthesis of selenoproteins in tissues including the mammary gland, with potential implications for milk quality. Literature is very limited about the effect of dietary selenium supplementation on selenoamino acids, only Sun et al [14] reported that yeast-selenium increased milk SeMet and Sec.

The antioxidant effects of selenium are primarily associated with the GPx family and TrxR. The GPx members use glutathione to catalyze the reduction of hydrogen peroxide and organic hydrogen peroxide and to protect cell membrane structure and function [22]. The TrxR catalyzes the reduction of oxidized thioredoxin, hydrogen peroxide, lipid hydroperoxides, and dehydroascorbate [23]. Literature regarding the effect of nanoSe on the activity of selenoenzymes in cows is unavailable. In lambs, nanoSe was effective in increasing selenium concentrations in blood, red blood cells, platelets, and the activity of GPx [24,25]. In mice, the enhancement of selenoenzyme activity contributed to nanoSe’s ability to scavenge free radicals, thereby contributing to growth, serum antioxidation, and selenium retention in the body [26]. Foroughi et al [27] reported that SS increased the transcript levels of DIO1 and DIO2 in ovine and bovine fetal thyrocytes *in vitro*. In this study, the activities of GPx4, GR, TrxR, and GST, and DIO were increased in milk for nanoSe diet, indicating that dietary nanoSe might promote the antioxidant defence system of peripartal dairy cows. This is particularly important given the increased oxidative stress associated with parturition and lactation.

Besides selenoenzymes, selenoproteins are involved in a variety of biological processes, including anti-aging, anti-inflammatory, anti-cancer, detoxification, maintaining cardiovascular health, and improving immunity and fertility [28]. In this study, dietary nanoSe upregulated the expression of a variety of selenoprotein genes, and only selenoprotein K was higher for nanoSe than SS. Han et al [11] showed that nanoSe upregulated the mRNA expression of GPx, TrxR, and selenoproteins W, T, K, and F, emphasizing the role of selenium as a key regulator of selenoprotein activity and expression in the mammary gland of dairy cows. In this study, the modulation of selenoproteins might not only enhance the antioxidant defense system but also contributes to the overall health and productivity of cows. The upregulation of selenoprotein in milk by nanoSe suggests a positive effect on the synthesis and secretion of these proteins, with the potential to improve milk quality and nutritional value. In addition, there were no effects of lactation weeks on milk selenium, selenoamino acids, selenoenzymes, and selenoproteins, indicating that these parameters were not changed with the progress of lactation weeks. This may be ascribed to the three-week addition of SS or nanoSe before lactation which led to a stable point for selenization in dairy cows.

## CONCLUSION

In contrast to SS, nanoSe showed greater effects on milk yield, selenium content, selenoamino acids contents, and selenoenzymes activity in milk. It is concluded that dietary nanoSe can be used to improve milk production and the milk profiles of selenoamino acids, selenoenzymes, and selenoproteins of dairy cows during the peripartal period.

## Figures and Tables

**Table 1:** Ingredients and nutrient levels of diets (DM basis) for peripartal dairy cows

Item	Prepartum	Postpartum
Ingredients (g/kg)		
Soy meal	121.5	134.9
Corn silage	578.0	256.2
Corn	73.1	116.2
Alfalfa hay	179.0	447.7
Wheat bran	38.9	35.5
Limestone	2.4	2.4
Ca_2_(PO_4_)_2_	2.2	2.2
NaCl	2.4	2.4
Premix^1)^	2.5	2.5
Nutrients (%)		
NE_L_^2)^	6.00	6.30
CP	14.14	17.01
NDF	45.70	33.10
peNDF	40.52	53.08
ADF	25.01	43.17
Ash	6.38	6.43
Ca	0.55	0.61
P	0.41	0.44

1) Supplied per kilogram of diet: vitamin D3 30,000 IU, vitamin A 200,000 IU, vitamin E 4,000 mg, niacin 3,900 mg, Fe 1,500 mg, I 0.3 mg, Cu 1,000 mg, Zn 4,000 mg, Mn 1,200 mg, I 50 mg, and Co 30 mg.

2) NEL (MJ/kg) and peNDF were calculated according to the Feed Database in China, and other values were determined.

CP, crude protein; NDF, neutral detergent fiber; peNDF, physically effective neutral detergent fiber; ADF, acid detergent fiber.

**Table 2:** Information of primers for quantitative real-time PCR

Name	GenBank	Primers (5’→3’)	Length (bp)	Efficiency (%)
SelenoP	NM_174459.2	F: aaggaagcaagctggtcgg R: actcgcaggtcctccaatct	263	98
SelenoT	NM_001103103.1	F: aaggggctggctcgtttaag R: aaacacccgcctgtaacctc	185	99
SelenoW	NM_174459.2	F: ctccaagaagggaggcgatg R: aagaccaggggtgtccatct	243	98
SelenoS	NM_001046114.2	F: acgtgtactaggggcctgc R: atgtaccagccgtaagtggc	197	97
SelenoF	NM_001034759.1	F: agggagatgcctgatgtgag R: agacgggcattaactacggg	113	98
SelenoM	NM_001163171.2	F: actggaaccgtctacaaggc R: ggtcatgtcgctgagtggaa	215	98
SelenoN	NM_001114976.2	F: ccgccatcagcgacttctat R: ccactccatgtccacgttca	200	96
SelenoK	NM_001037489.2	F: gttggacagcaggagtcagt R: cagatgagcttccgtagcct	143	99
SelenoO	NM_001163193.2	F: atgtacttgggcgaggtgtg R: tccaaacctgaggaacgtgg	294	98
SelenoV	NM_001163244.1	F: actccattggccaccgattt R: aggccacagtaaaccactcg	224	97
SelenoH	NM_001164092.1	F: gcacgagctgacgagtctac R: cagaggctcagcgagacg	283	98
SelenoI	NM_001075257.1	F: gggacagagactccacttgc R: ttactggcggacccaaagtc	161	97
Beta-actin	AY141970.1	F: caaccgtgagaagatgaccca R: tgtcacggacgatttccgctc	293	99

PCR, polymerase chain reaction; bp, base pair.

**Table 3:** Effect of nanoSelenium on the milk production of peripartal dairy cows

Items	Dietary treatments^1)^			SEM	p-values		
	
	Control	SS	nanoSe		Treatment	Week	Interaction
Cows	15	15	15				
DMI (kg/d)	15.7	16.1	16.1	0.110	0.053	0.051	0.321
Milk yield (kg/d)	23.6^b^	24.1^b^	25.9^a^	0.182	<0.001	<0.001	0.002
Milk yield/DMI	1.50^b^	1.50^b^	1.61^a^	0.033	0.026	0.041	0.105
Milk fat (%)	3.00	3.07	3.04	0.061	0.735	<0.001	0.001
Milk protein (%)	3.02^b^	3.14^ab^	3.22^a^	0.049	0.027	<0.001	0.124
Milk lactose (%)	4.01	3.90	3.99	0.046	0.226	<0.001	0.018
Milk total solids (%)	12.0	11.9	12.1	0.095	0.306	<0.001	0.011
FCM (kg/d)	20.5^b^	20.3^b^	22.2^a^	0.257	<0.001	<0.001	0.001

1) Control, background selenium at 0.06 mg/kg; SS, supplemental selenium from sodium selenite at 1.00 mg/kg; nanoSe, supplemental selenium from nanoSeleinium at 1.00 mg/kg.

a,b Mean values in the same row with different superscripts differ (p<0.05).

SEM, standard error of the mean; SS, sodium selenite; nanoSe, nanoselenium; DMI, dry matter intake; FCM, fat-corrected milk.

**Table 4:** Effect of nanoselenium on the milk profiles of selenium, selenoamino acids, and selenoenzymes in peripartal dairy cows

Items	Dietary treatments^1)^			SEM	p-values		
	
	Control	SS	nanoSe		Treatment	Week	Interaction
Cows	15	15	15				
Contents (μg/L of milk)							
Selenium	37.4^c^	143^b^	207^a^	0.802	<0.001	0.051	0.521
Sec	28.2^c^	93.8^b^	135^a^	4.120	<0.001	0.054	0.196
SeMet	74.2^c^	227^b^	328^a^	6.081	<0.001	0.115	0.335
DIO2	21.9^c^	33.0^b^	49.8^a^	0.939	<0.001	0.131	0.524
DIO3	93.1^c^	189^b^	248^a^	6.083	<0.001	0.102	0.421
Activity (U/g of milk protein)							
GPx4	24.3^b^	27.4^b^	37.5^a^	1.043	<0.001	0.126	0.137
GR	21.8^c^	25.7^b^	45.8^a^	1.015	<0.001	0.213	0.226
TrxR	18.0^c^	21.2^b^	26.7^a^	0.988	<0.001	0.115	0.058
GST	13.0^c^	17.5^b^	24.5^a^	0.917	<0.001	0.220	0.145

1) Control, background selenium at 0.06 mg/kg; SS, sodium selenite, supplemental selenium from sodium selenite at 1.00 mg/kg; nanoSe, supplemental selenium from nanoSe at 1.00 mg/kg.

a–c Mean values in the same row with different superscripts differ (p<0.05).

SEM, standard error of the mean; SS, sodium selenite; nanoSe, nanoselenium; Sec, selenocysteine; SeMet, selenomethionine; DIO, odothyronine deiodinase; GPx, glutathione peroxidase; GR, glutathione reductase; TrxR, thioredoxin reductase; GST, glutathione S-transferase.

**Table 5:** Effect of nanoselenium on the mRNA expression (2^−ΔΔCt^) of selenoproteins in the milk of peripartal dairy cows

Items	Dietary treatments^1)^			SEM	p-values		
	
	Control	SS	nanoSe		Treatment	Week	Interaction
Cows	15	15	15				
SelenoP	0.251^b^	0.385^a^	0.401^a^	0.0055	<0.001	0.230	0.208
SelenoT	0.099	0.104	0.105	0.0017	0.098	0.135	0.442
SelenoW	0.162^b^	0.171^a^	0.176^a^	0.0018	<0.001	0.147	0.046
SelenoS	0.193^b^	0.213^a^	0.214^a^	0.0024	<0.001	0.131	0.159
SelenoF	0.009^b^	0.010^ab^	0.011^a^	0.0004	<0.001	0.054	0.068
SelenoM	0.293^b^	0.332^a^	0.336^a^	0.0045	<0.001	0.157	0.072
SelenoN	0.197^b^	0.259^a^	0.272^a^	0.0040	<0.001	0.214	0.195
SelenoK	0.168^c^	0.186^b^	0.201^a^	0.0041	<0.001	0.163	0.362
SelenoO	0.184^b^	0.190^a^	0.195^a^	0.0016	<0.001	0.178	0.222
SelenoV	0.050	0.054	0.054	0.0012	0.127	0.339	0.567
SelenoH	0.030^b^	0.032^a^	0.033^a^	0.0005	<0.001	0.241	0.320
SelenoI	0.028^b^	0.031^a^	0.031^a^	0.0005	<0.001	0.119	0.137

1) Control, background selenium at 0.06 mg/kg; SS, supplemental selenium from sodium selenite at 1.00 mg/kg; nanoSe, supplemental selenium from nanoSe at 1.00 mg/kg.

a–c Mean values in the same row with different superscripts differ (p<0.05).

SEM, standard error of the mean; SS, sodium selenite; nanoSe, nanoselenium.
